# Holobiont responses of mesophotic precious red coral *Corallium rubrum* to thermal anomalies

**DOI:** 10.1186/s40793-023-00525-6

**Published:** 2023-08-14

**Authors:** Romie Tignat-Perrier, Jeroen A. J. M. van de Water, Denis Allemand, Christine Ferrier-Pagès

**Affiliations:** 1 Unité de Recherche sur la Biologie des Coraux Précieux CSM-CHANEL, Centre Scientifique de Monaco, 8 Quai Antoine 1er, 98000 Monaco, Principality of Monaco; 2 Coral Ecophysiology Laboratory, Centre Scientifique de Monaco, 8 Quai Antoine 1er, 98000 Monaco, Principality of Monaco; 3 Department of Estuarine and Delta Systems, Royal Netherlands Institute for Sea Research, Korringaweg 7, 4401 NT Yerseke, The Netherlands; 4 Centre Scientifique de Monaco, 8 Quai Antoine 1er, 98000 Monaco, Principality of Monaco

**Keywords:** Red coral, *Corallium rubrum*, Octocoral, Gorgonian, Thermal stress, Bacterial communities, Physiology, Holobiont, Gene expression, *16S rRNA* gene sequencing

## Abstract

Marine heat waves (MHWs) have increased in frequency and intensity worldwide, causing mass mortality of benthic organisms and loss of biodiversity in shallow waters. The Mediterranean Sea is no exception, with shallow populations of habitat-forming octocorals facing the threat of local extinction. The mesophotic zone, which is less affected by MHWs, may be of ecological importance in conservation strategies for these species. However, our understanding of the response of mesophotic octocoral holobionts to changes in seawater temperature remains limited. To address this knowledge gap, we conducted a study on an iconic Mediterranean octocoral, the red coral *Corallium rubrum* sampled at 60 m depth and 15 °C. We exposed the colonies to temperatures they occasionally experience (18 °C) and temperatures that could occur at the end of the century if global warming continues (21 °C). We also tested their response to extremely cold and warm temperatures (12 °C and 24 °C). Our results show a high tolerance of *C. rubrum* to a two-month long exposure to temperatures ranging from 12 to 21 °C as no colony showed signs of tissue loss, reduced feeding ability, stress-induced gene expression, or disruption of host-bacterial symbioses. At 24 °C, however, we measured a sharp decrease in the relative abundance of Spirochaetaceae, which are the predominant bacterial symbionts under healthy conditions, along with a relative increase in Vibrionaceae. Tissue loss and overexpression of the tumor necrosis factor receptor 1 gene were also observed after two weeks of exposure. In light of ongoing global warming, our study helps predict the consequences of MHWs on mesophotic coralligenous reefs and the biodiversity that depends on them.

**Supplementary Information:**

The online version contains supplementary material available at 10.1186/s40793-023-00525-6.

## Introduction

Global warming is one of the main drivers of marine biodiversity loss worldwide [1–5] and particularly in the Mediterranean Sea, which is one of the climate change ‘hotspots’ because of its rapid response to atmospheric forcing [6–8]. Marine heat waves (MHWs) are characterized by prolonged periods of anomalously high sea surface temperatures, and have increased in intensity and frequency in recent decades. In 2022, all surface waters of the western Mediterranean region were exposed to at least one MHW during the period from May to August 2022. During this period, sea surface temperature anomalies were locally 4–5 °C above the summer average, exceeding the previous maximum of +2–3 °C during the 2003 MHW [9]. Recurrent MHWs have led to outbreaks of microbial diseases and mass mortalities affecting the ecosystem engineer species of the coralligenous reefs, which are among the most biodiverse assemblages in the Mediterranean Sea [10–13].

Corals are the keystone species of these ecosystems, they increase the overall complexity of the benthic ecosystem and provide habitat for many species [14–16]. Therefore, the decline in coral populations because of MHWs can disrupt the intricate balance of the ecosystem and negatively affect the associated biodiversity. Among the Mediterranean corals, the slow-growing, long-lived red coral species *Corallium rubrum* plays a prominent economic role, because it harbors a hard and intensely red-colored skeleton that has been of religious and cultural importance since ancient times, and is stillused in jewelry. Over-harvesting, in conjunction with recent MHWs, is actively contributing to reduced populations of red coral in shallow waters (< 30 m depth) [11, 12, 17, 18], which has resulted in this species being listed as Endangered by the International Union for Conservation of Nature (IUCN red list [19]). Shallow-water colonies do not reach large sizes and are often immature and not fully reproductive (sexual maturity at 10 years of age) in contrast to mesophotic (> 30 to 150 m depth) colonies, which have been preserved from overfishing and temperature extremes (i.e., up to 18 °C for a few days at 50 m depth) [20–23]. Currently, mesophotic populations are exposed to a relatively stable thermal regime throughout the year and few mortality events associated with MHWs below 50 m depth have been observed [24, 25]. Because mesophotic coral ecosystems are difficult to access [23], few data are available on the biology and environmental factors that may pose a threat to these populations [24–29]. However, projections for future climate change indicate that mesophotic populations may soon be exposed to temperatures they have never or rarely experienced [30, 31]. It is therefore imperative to assess the survival prospects of mesophotic populations of *C. rubrum* under climate change projections.

To this date, only four experimental studies have assessed the physiological response of *C. rubrum* to heat stress, most of which focused on shallow populations, which are thought to have better thermotolerance due to their thermal history [32–35]. Although they showed that corals experienced a stress at temperatures above 24 °C, these investigations were unable to identify potential factors of resilience to heat stress. In tropical coral species, microbial symbionts have been found to contribute to stress tolerance and disease resistance by producing antioxidants, providing nutrients and protecting the animal host from pathogens [36–39]. Consequently, numerous studies have investigated the changes in the bacterial communities associated with tropical corals affected by MHWs and/or thermal stress [40–47], but few have investigated this regarding Mediterranean temperate corals [25, 48, 49]. *C. rubrum* stands out for its specific microbiota dominated by bacteria from the Spirochaetaceae family [50, 51], while *Endozoicomonas* dominate the bacterial communities associated with the most Mediterranean coral species [49, 51–53] as well as many tropical corals [36, 54–56]. The symbiosis between *C. rubrum* and Spirochaetaceae appears to be stable across seasons and geographic locations [50, 51], suggesting that Spirochaetaceae may play a crucial role in the overall health of the holobiont. However, to our knowledge, just one study has examined the response of bacterial communities associated with *C. rubrum* under thermal stress [25]. This ecological field study was conducted during the 2011 MHW and found that the bacterial community of shallow populations exposed to 3–4 °C than usual summer temperatures had a similar microbiome structure as those from a similar population in 2017 in the absence of stressors [25]. In contrast, mesophotic corals had lower relative abundances of Spirochaetaceae while Vibrionaceae were more abundantly present, despite being exposed to much lower temperatures [25], always in comparison with shallow populations studied in 2017 [50]. Taken together, we are still lacking a comprehensive understanding of how the red coral holobiont may be affected by thermal stress. Holistic studies monitoring coral health, physiology and microbiota under controlled conditions are thus needed to better understand the response of *C. rubrum* to temperature anomalies.

In this study, we examined the response of mesophotic colonies of *C. rubrum* to a wide range of thermal conditions, including unusually cold and warm temperatures (i.e., five thermal conditions ranging from 12 to 24 °C) in a controlled experiment. By assessing both the bacterial community composition and host physiological status (i.e., (partial) mortality/survival, energy reserves and expression of stress response genes), we aimed to relate temperature changes to the response of the holobiont and identify temperatures that may be detrimental to mesophotic populations of *C. rubrum*.

## Material and methods

### Biological material and annual thermal regime at collection site

Thirty-six colonies of *C. rubrum* (7 cm long branched colonies) were collected in June 2021 at 60 m depth in Villefranche-sur-Mer (France), with the persmission of the Direction Inter-régionale de la Mer Méditerranée (France). The seawater temperature near the colonies at the time of collection was 15 °C according to the divers’ computers. At this site, seawater temperature remained broadly stable, fluctuating between 13.5 and 15.5 °C over the past two years, occasionally reaching ≥ 18 °C in autumn (Additional file 1: Fig. S1). Temperatures ≥ 18 °C at this depth occur only transiently (duration of less than one week) in the fall following the seasonal mixed layer depth (MLD) deepening [57, 58]. Colonies were brought back to the aquarium facility of the Centre Scientifique de Monaco, where they were divided into two fragments with a length of ~ 5 cm. Nubbins were hanged on a thread of fishing line and kept in open flow aquaria at 15 °C in the dark for two weeks, before being randomly distributed over the experimental aquaria as described below.

### Experimental design

To evaluate the impact of thermal stress on the physiology and microbiota of *C. rubrum*, colonies were exposed to five thermal conditions. In a first experiment, colonies were either maintained at 15 °C throughout the experiment or gradually changed (± 0.5 °C every two days) to 12 °C, 18 °C or 21 °C and maintained under these thermal conditions for eight weeks. Temperatures from 12 to 18 °C were selected because they cover the entire range of temperatures that *C. rubrum* experiences at 60 m depth. Although 12 °C is a relatively low temperature for the upper-mesophotic zone (such as 60 m depth), populations exposed to upwelling events in fall, or those found in the lower mesophotic zone may commonly experience this temperature. A treatment of 21 °C was included as this temperature may be reached in the mesophotic zone at the end of the century if global warming continues [30, 31]. The first experiment was followed by a second experiment, in which red coral colonies were either maintained at 15 °C throughout the experiment or were gradually (+0.5 °C every two days) exposed to 24 °C. This temperature is common for shallow populations of *C. rubrum* in summer (Additional file 1: Fig. S1), and we aimed to test the upper limit of thermotolerance for mesophotic populations of *C. rubrum*. Colonies were maintained under these thermal conditions for two weeks, when colonies at 24 °C suffered tissue loss.

The setup of the first and second experiment consisted of, respectively, twelve and six 25 L aquaria that were kept in the dark for the duration of the experiment. The aquaria received a continuous flow (15 L/h) of seawater that was pumped from 50 m-depth, and filtered through a 5 µm filter, a charcoal filter and treated with UV light to eliminate microorganisms. In each aquarium, a pump ensured continuous seawater mixing and a heater, controlled by a temperature-controller, maintained a constant seawater temperature. *C. rubrum* fragments were fed with fresh *Artemia salina* nauplii, and the aquaria were cleaned once a week.

Three aquaria were assigned to each thermal condition. The fragments were then randomly distributed over the aquaria (36 fragments for first experiment and 18 fragments for second experiment), resulting in three fragments per aquarium, and kept at 15 °C for two weeks to acclimate. Sampling was done at the end of the experiment for physiological and molecular analyses as detailed below.

### Host physiological parameters

#### Coral energetics

Samples of approximately 4 cm long were cut from each fragment and immediately flash frozen and stored at − 80 °C until further processing. Samples were lyophilized overnight (Christ Martin™ Alpha™ Freeze dryer, Fisher Scientific). The tissue was then removed from the skeleton using a sterile scalpel blade, ground into powder using a mortar and pestle, and the total dry weight (DW) of the tissue was determined. Subsamples with a known weight were used to perform the various analyses described below.

A sub-sample of approximately 10 mg was used to determine the ash free dry weight (AFDW) and the amount of sclerites (small CaCO_3_ structures embedded in the coenenchyma) per mg of DW. To this end, subsamples of each fragment were accurately weighed before and after being burned at 450 °C for 4 h. The AFDW was calculated from the difference between the total dry weight and the ash weight. The weight of the inorganic ash fraction was used as a proxy of the sclerite content of the tissue.

Proteins were extracted from 5 mg of dry tissue in 200 µL of 1 M sodium hydroxide and heated at 90 °C for 30 min. Total protein concentration was then measured using the Pierce BCA Protein Assay kit (Thermo Fisher Scientific) following the manufacturer’s instructions and expressed in mg of protein per mg AFDW.

To extract lipids, 10 mg of dry tissue were added to 1.5 mL of chloroform/methanol (2v:1v) in glass tubes and incubated at room temperature for 20 min on an orbital shaker. The tubes were centrifuged at 1000 g for 5 min to remove debris and 1 mL of extract was subsequently evaporated in a dry bath at 90 °C. Total lipid concentration was measured using a protocol based on the sulpho-phospho-vanillin reaction method from Barnes and Blackstock [59] in a 96-well microplate format [60]. The net absorbance values at 520 nm were read using a spectrofluorometer (Xenius^®^, SAFAS, Monaco) and were compared with a standard curve made of known concentrations of cholesterol. Total lipid concentrations were expressed in mg of lipids per mg AFDW.

Carbohydrates were quantified using the method of Dubois et al. [61], using D-glucose as standard. Five mg of lyophilized tissue were mixed with 1 mL of 5% phenol and 5 mL of sulfuric acid in glass tubes, and incubated for 30 min (10 min at RT, then 20 min at 30 °C). The tubes were centrifuged at 5000 g for 5 min to remove debris, and 300 µL of extract was transferred to a 96-well microplate for colorimetric detection (net absorbance values read at 490 nm). Carbohydrate concentration was expressed in µg per mg AFDW.

#### Extraction of nucleic acids

Coral samples of 0.5 cm long were taken from each fragment and preserved in RNAlater^®^ RNA Stabilization Solution (Qiagen) at 4 °C until further processing. Total RNA and DNA were extracted using the AllPrep^®^ DNA/RNA Micro kit (Qiagen) with the following modifications: samples were transferred in pre-filled bead tubes (Qiagen) containing 346.5 µL of Buffer RLT Plus and 3.5 µL of beta-mercaptoethanol, followed by 2 times 2 min of bead beating using the CryoMill (Retch, Germany) at a frequency of 30 Hz. One negative extraction control sample (i.e., extraction without sample material) was processed at the same time as the samples of red coral in order to account for contaminants. The quality (RNA Integrity Number > 7) and concentration of the purified RNA were evaluated on a Bioanalyzer using the Agilent RNA 6000 Nano kit, and RNA was stored at − 80 °C.

#### Gene expression of heat stress protein 70 (HSP70) and tumor necrosis factor receptor 1 (TNFR1)

qPCR primers to amplify the *TNFR1* and beta actin genes were designed using Primer3 (http://primer3.sourceforge.net/) and the red coral transcriptome (P. Ganot, unpublished data), while primers to amplify the *HSP70* gene were taken from Haguenauer et al. [34]. The newly designed primers were verified and evaluated for primer specificity through a gel electrophoresis and melting curve analysis of the amplification product obtained with a mix of *C. rubrum* cDNA as a template. Efficiency was verified by doing an amplification of a series of twofold dilutions of *C. rubrum* cDNA covering two orders of magnitude of template amount. The results were plotted as C_t_ versus log_2_[cDNA], and the primer-specific amplification efficiency *E* was derived from the slope of the regression using formula *E* = 2^−(1/slope)^ [62]. Primer pairs with *E* outside 1.85–2.15 range were redesigned and re-validated. Final primer sequences are given in Additional file 1: Table S1 and the descriptive statistics (R-package *ctrl* [63], *cpSta* function) on the reference *beta actin* gene expression are presented in Additional file 2.

For the reverse transcription qPCR analyses, cDNA was synthesized from 10 ng of total RNA in a final reaction volume of 20 µL using the SuperScript IV Reverse Transcriptase (ThermoFisher). Quantitative PCR (qPCR) reactions were performed in a total volume of 10 µL with 1:20 dilution of cDNA template aliquots and 1 µM of primers using SYBR-green-based detection (SensiFAST™ SYBR^®^ No-ROX kit, ThermoFisher) on a QuantStudio3 qPCR machine (Applied Biosystems). Cycle parameters were 95 °C 5 min then 40 cycles 95 °C 10 s/60 °C 30 s. Following amplification, the specificity of the product was assessed from a melting curve program. Delta cycle threshold (dCt) values were calculated by subtracting the Ct of the reference gene (beta actin gene) from the Ct of the genes of interest. The delta delta Ct (ddCt) values were calculated by subtracting the dCt of the treatment samples from the dCt of the control samples, and fold changes were calculated using the 2^−ddCt^ method [64].

#### Data analyses

All graphical and statistical analyses were carried out in the R environment (version 4.2.2). Generalized linear mixed effects models (R-package *lme4*) [65] were fitted including ‘thermal condition’ as a fixed factor and ‘experimental tank’ as a random factor to assess whether a tank effect impacted the physiological parameters (protein, lipid and carbohydrate content per mg of AFDW) and gene expressions (fold change values). As no significant random effects were observed, all data were analyzed using mixed-effects linear models (R-package *lme4*) with ‘thermal condition’ as fixed factor. Normality and homoscedasticity of the residuals were verified using the Shapiro–Wilk and Levene’s tests (R-package *car*) [66], respectively. In case residuals did not follow a normal distribution, a Box-Cox transformation was applied prior to model fitting.

### Bacterial community analysis

#### DNA extraction and sequencing

DNA was simultaneously extracted with total RNA from the samples using the AllPrep^®^ DNA/RNA Micro kit (Qiagen) with modifications as detailed above. Seawater, *Artemia salina* and negative control (i.e., extraction without sample material) samples were processed at the same time as the samples of *C. rubrum*. For seawater samples, one liter of seawater per thermal condition was filtered on a 0.2 µm polyethersulfone (PES) filter before DNA extraction. For the *Artemia salina* sample, DNA was extracted from 10 mg of fresh organisms. DNA concentration was measured using an Invitrogen™ Qubit™ 4 Fluorometer (Fisher Scientific) and the Qubit^®^ dsDNA BR (Broad-Range) Assay kit, and DNA was stored at − 20 °C. DNA was then sent to STAB-VIDA (Portugal) for amplicon library preparation using Illumina’s standard “16S Metagenomic Sequencing Library Preparation” protocol (Illumina, 2013). The V3-V4 region of the *16S rRNA* gene was amplified using the forward primer 341F 5’-CCTACGGGNGGCWGCAG-3’ and the reverse primer 785R 5'-GACTACHVGGGTATCTAATCC-3’ [67]. For some samples, library preparation failed due to insufficient amplification during the first PCR step, and these samples were thus not included in the pooled library (Additional file 1: Table S2). Libraries were pooled in equimolar ratios and paired-end (2 × 300 bp) sequenced on the Illumina MiSeq platform with V3 chemistry. The samples from the two experiments were processed on two different lanes. The fastq files containing the raw sequencing data have been deposited in the NCBI’s Short Read Archive (SRA) under the BioProject accession number PRJNA967137.

#### Bioinformatics data processing

The *16S rRNA* gene amplicon data were processed using the DADA2 pipeline (version 1.16) [68]. MiSeq sequencing produced 3,618,883 reads, ranging from 2350 to 122,538 reads per sample (Additional file 1: Table S2). Forward and reverse reads were trimmed and filtered with the following settings: truncLen = c(250, 230), maxN = 0, maxEE = c(2, 2), rm.phix = TRUE, trimLeft = c(17, 21). Error rates were computed and used for sequence inference. Sequences were merged, and those smaller than 390 bp and longer than 450 bp were removed, and an Amplicon Sequence Variant (ASV) count table was created. Chimeras were checked and removed. The number of reads passing the different steps of the pipeline per sample is presented in Additional file 1: Table S2. Taxonomy was assigned to the 3778 ASVs using the SILVA SSU reference database and a minimum bootstrap confidence of 50 (version 138.1). The ASV table, the ASV taxonomy, the sequences of each ASV and the metadata are available as Additional file 2 (Additional file 3, Additional file 4, and Additional file 5).

#### Data analyses

The R-package *decontam* [69] was used to identify potential contaminant ASVs in the samples based on the negative control sample (*isContaminant* function). Two ASVs were identified as potential contaminant and were removed. Chao1 estimates of species richness and evenness (Shannon index) were calculated using the R-package *vegan* [70] on the ASV count table, and analyses of variance (ANOVA) were used to test whether species richness and evenness were similar between thermal conditions. To examine changes in the composition of *C. rubrum* microbiome, we used a compositional data analysis (CoDA) [71]. Additional file 6 were transformed by calculating the centered log-ratios (clr) as implemented in the R-package *compositions* [72]*,* after imputing zero counts based on Bayesian multiplicative replacement (Bayes-LaPlace BM method of the *cmultRepl* function of the R-package *zCompositions*) [73]. Clr-transformed count data were then used as inputs for multivariate hypothesis testing [71, 74]. An Aitchison distance matrix was generated by calculating the Euclidean distances between samples based on the clr-transformed data table. Based on the Aitchison distance matrix, hierarchical cluster analyses (WardD2 method), principal component analyses (PCA), as well as permutational multivariate analysis of variance (perMANOVA/adonis and permanova_pairwise; R-package *vegan* and *ecole* [75]) and dispersion analyses (PERMDISP; R-package *vegan*) were used to assess differences in bacterial community diversity and dispersion between the thermal conditions.

Differential abundance analyses were performed to identify ASVs that differed in abundance between thermal conditions. For this purpose, the R-package *ANCOM-BC* (*ancombc2* function, version 02-2023) [76] was used, excluding ASVs with a proportion of zero greater than 0.2 and 0.4 for the first and second experiment respectively. ASVs were considered differentially abundant if α < 0.05. To gain further insight into the origin (i.e., coral, seawater or *Artemia salina* nauplii) of the differentially abundant ASVs found in high relative abundance in a given thermal condition compared to the 15 °C control condition, their presence in the seawater samples collected around the colonies or in the *Artemia salina* food was assessed. For some ASVs, the species assignment was verified by blasting the ASV sequence on the *nr* database using the blastn algorithm [77].

Distance-based redundancy analyses (dbRDA) were also carried out to evaluate whether there were relationships between physiological parameters of the coral host (i.e., protein, lipid and carbohydrate content) and bacterial community structure.

## Results

### Host response to temperature

At the end of the first experiment (eight weeks of incubation at 12 °C, 15 °C, 18 °C and 21 °C), the sclerite content was on average higher at 21 °C compared to the 15 °C control condition (Table 1; *P* = 0.05). No significant difference in the energy reserves (protein, lipid and carbohydrate content per mg AFDW) or in mRNA levels of *TNFR1* and *HSP70* was detected between temperature conditions (Table 1 and Additional file 2). No sign of tissue damage or loss was recorded.

**Table 1:** Effect of different thermal conditions on the biomass (protein, lipid and carbohydrate content) and expression of stress response genes in fold change (*TNFR1* and *HSP70*)

*Physiology*						
Experiment	Temperature	Proteins (mg per mg AFDW)	Lipids (mg per mg AFDW)	Carbohydrates (μg per mg AFDW)	Sclerites (% dry tissue)	Tissue loss
N°l	15 °C—Control	5848.0 ± 542.8	136.6 ± 44.3	203.5 ± 58.9	68.9 ± 4.6	No
	12 °C	6271.8 ± 646.1	142.4 ± 32.7	256.9 ± 47.5	68.9 ± 4.1	No
	18 °C	6143.3 ± 1074.4	128.4 ± 47.8	242.4 ± 75.5	73.9 ± 4.0	No
	21 °C	5616.9 ± 595.2	131.6 ± 99.6	295.2 ± 48.8	74.6 ± 6.5	No
N°2	15 °C—Control	5144.6 ± 350.7	127.4 ± 43.0	461.5 ± 142.1	72.8 ± 7.0	No
	24 °C	5474.6 ± 296.2	68.6 ± 32.3	392.2 ± 58.7	80.0 ± 3.7	Yes

*Gene expression*			
Experiment	Temperature	*TNFR1* gene expression (fold change)	*HSP70* gene expression (fold change)
N°l	15 °C—Control	1 ± 0	1 ± 0
	12 °C	1.3 ± 0.52	0.93 ± 0.57
	18 °C	0.92 ± 0.68	1.9 ± 1.1
	21 °C	1.5 ± 1.01	0.77 ± 0.38
N°2	15 °C—Control	1 ± 0	1 ± 0
	24 °C	5.1 ± 2.9	2.2 ± 1.3

In the second experiment, *C. rubrum* fragments were exposed to 15 °C or 24 °C for only two weeks, as corals maintained at 24 °C showed substantial tissue loss, leading to partial colony mortality of ≥ 30% of the surface (Additional file 1: Fig. S2). However, no significant differences were detected between the energy reserves in the live tissues (protein, lipid and carbohydrate content per mg AFDW) of corals kept at 24 °C and 15 °C (Table 1 and Additional file 2). But the sclerite content was on average higher in corals at 24 °C compared to 15 °C (*P* = 0.02). In addition, the expression of the *TNFR1* gene was significantly higher in corals exposed to 24 °C than those maintained at 15 °C (fold change of 5.1; *P* = 0.001), whereas no differences were found in the expression of *HSP70* (Table 1 and Additional file 2).

### Response of the bacterial communities of *C. rubrum* to temperature variations

**Fig. 1:**
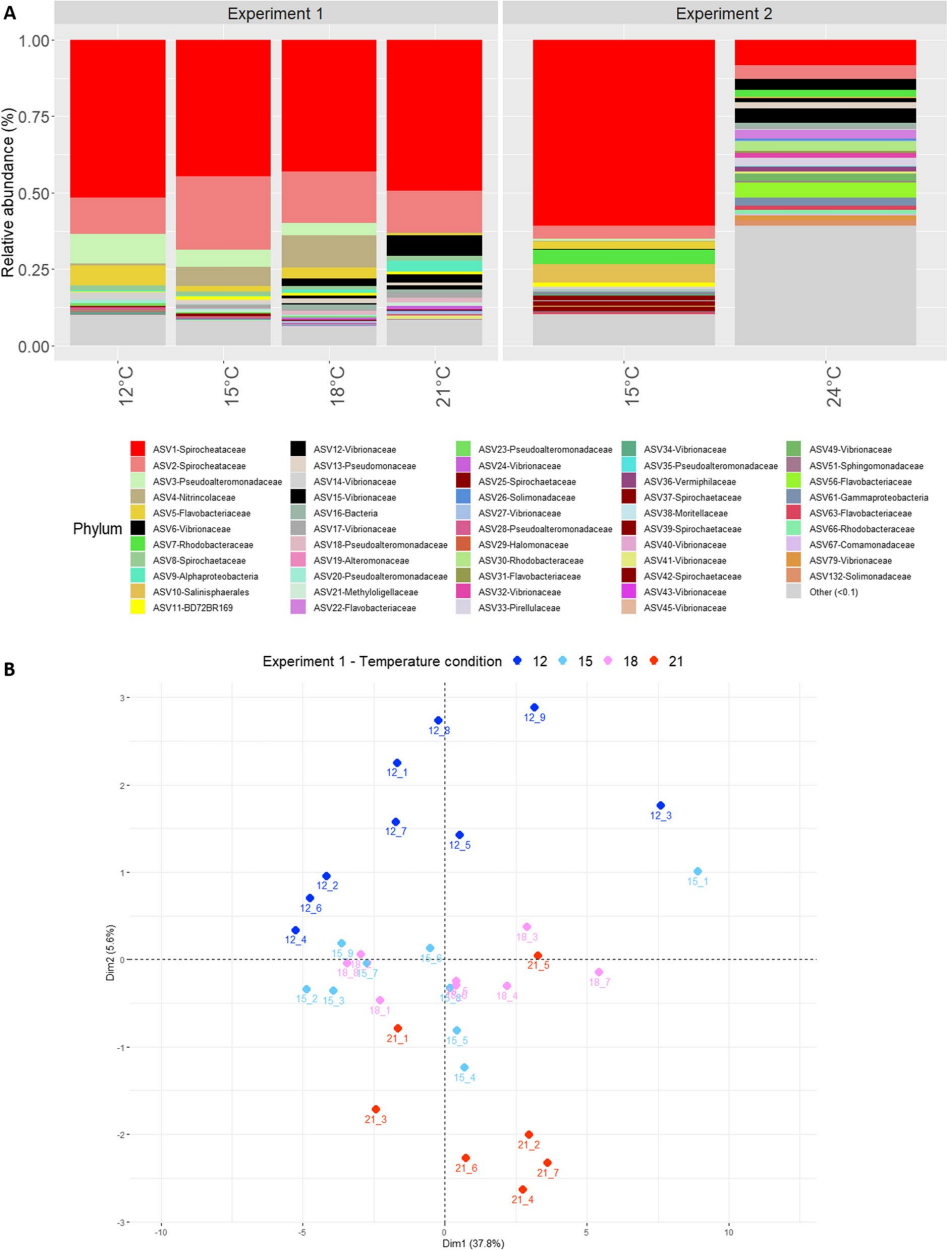
Impact of the different temperature conditions on the structure of the bacterial communities of *Corallium rubrum*. **A** Relative abundance of the most abundant ASVs under the different temperature conditions in both experiments (Other include ASVs representing < 10% of the dataset); **B** Principal component analysis of the Aitchison distance matrix based on the composition of the bacterial community (ASV level) associated with *C. rubrum* exposed to different temperatures (12 °C, 15 °C, 18 °C and 21 °C, Experiment 1)

Overall, the composition of the bacterial communities was structurally different between *C. rubrum* and seawater samples (*P* = 0.005; Additional file 1: Fig. S3). The most abundant bacterial ASVs in *C. rubrum* were ASV1-Spirochaetaceae (43%), ASV2-Spirochaetaceae (13%), ASV3-Pseudoalteromonadaceae (3%) and ASV4-Nitrincolaceae (2%), although their relative abundance varied between thermal conditions (Fig. 1A and Additional file 1: Fig. S4).

**Fig. 2:**
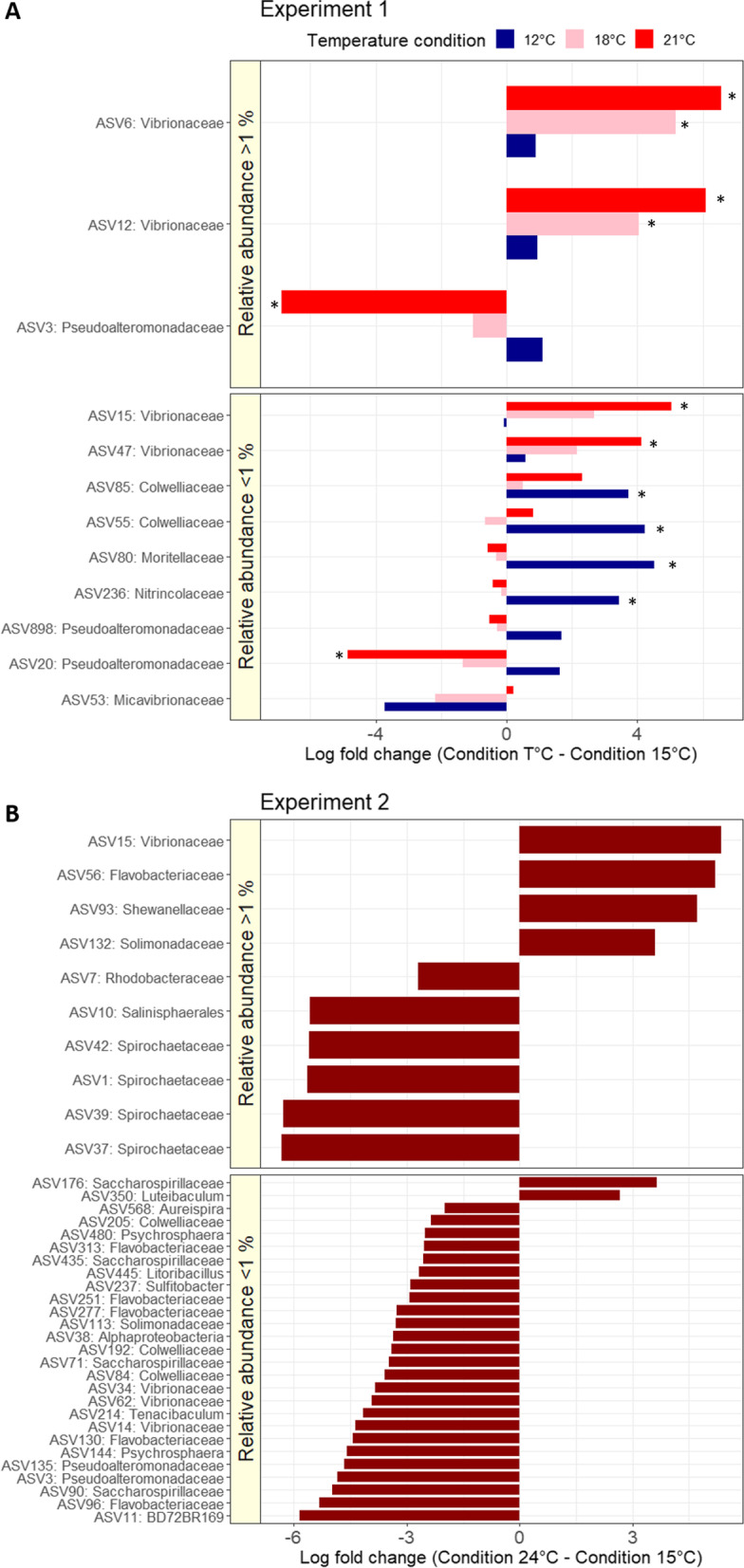
Bacterial ASVs differentially abundant between temperature conditions (log fold change—natural log). ASVs differentially abundant in one temperature condition compared to the 15 °C control temperature in the first (**A**; stars indicating significant changes) and second (**B**) experiment. The ASVs were separated in two groups depending on their average relative abundance: ≥ 1% and < 1% (i.e., ‘rare’ ASVs)

In the first experiment, the bacterial community composition of *C. rubrum* differed significantly among temperatures (12 °C, 15 °C, 18 °C and 21 °C; PERMANOVA multiple comparisons *P* < 0.05; Fig. 1B and Additional file 2), whereas the species richness (Chao1 estimator) and evenness (Shannon index) did not differ among conditions (Additional file 1: Fig. S5 and Additional file 2). Differential abundance analyses identified six ASVs whose abundance changed between the 15 °C and 21 °C conditions (Fig. 2A and Additional file 1: Fig. S6). The abundances of ASV6 and ASV12 belonging to the genus *Vibrio* both significantly increased (from 0.003% at 15 °C to 5.2% at 21 °C on average), whereas the relative abundance of ASV3 belonging to the genus *Pseudoalteromonas* decreased (from 5% at 15 °C to 0.01% at 21 °C). Those ASV6 and ASV12 are *Vibrio sp*. most closely related to the pathogens *V. orientalis* and *V. fortis*.

Vibrionaceae ASVs which were present at relatively higher abundances in the bacterial communities of *C. rubrum* colonies exposed to 18 °C and 21 °C were also found in both the 21 °C seawater sample (with a low relative abundance of 0.001%) and the *Artemia salina* sample (with a high relative abundance of 1.9%; Additional file 1: Fig. S6). ASV3-*Pseudoalteromonas*, observed in higher relative abundance in the bacterial communities of *C. rubrum* maintained at 12 °C, was detectable in the corresponding seawater sample but not in the *Artemia salina* sample.

Exposure of *C. rubrum* to 24 °C for two weeks during the second experiment resulted in significant changes in bacterial community composition compared to 15 °C (*P* = 0.02; Fig. 1A and Additional file 1: Fig. S4). Bacterial communities exhibited a higher evenness (Shannon index) at 24 °C than at 15 °C (*P* = 10^–5^), whereas Chao1 estimated richness did not change at 24 °C (*P* > 0.05; Additional file 1: Fig. S5 and Additional file 2). The *ANCOM-BC* analysis identified 37 differentially abundant ASVs between the two temperature conditions, including ten dominant ASVs (≥ 1%) (Fig. 2 and Additional file 1: Fig. S7). Four of those ASVs (i.e., ASV1, 37, 39 and 42) belonged to the Spirochaetaceae family, and were primarily responsible for the overall reduced relative abundance in Spirochaetaceae from 66% at 15 °C to 8% at 24 °C. In contrast, ASV15 from the genus *Vibrio* increased significantly in abundance at 24 °C (from 0.003% at 15 °C to 4.8% at 24 °C). This *Vibrio* ASV is most closely related to the pathogen *V. tubiashii*.

In both experiments, redundancy analyses (RDA) revealed no correlation between changes in bacterial communities and changes in physiology (biomass parameters and stress response gene expression) of red coral colonies under the different thermal conditions (*P* > 0.05).

## Discussion

### Tolerance of the host to abnormal thermal conditions

Shallow and upper-mesophotic populations of *C. rubrum* have been shown to withstand temperatures up to 24 °C without major effects on their physiological performance, such as oxygen consumption, respiration rate and polyp reactivity [32–35]. These populations live in environments with rapid temperature fluctuations and temperature extremes in summer, and thus tend to have a large thermotolerance range reflecting their environment [34, 35, 78, 79]. Thus, mortality has been observed in these shallow populations when temperatures reach 25 °C [32–35]. In contrast, mesophotic populations experience relatively stable (and lower) temperature regimes around 14–15 °C and are therefore expected to have a lower thermal tolerance than their shallow conspecifics. This has previously been observed in several tropical and temperate invertebrate species, because global warming has not yet acted as a genetic bottleneck that selects resistant individuals [78, 80]. Our results indeed suggest that mesophotic colonies of *C. rubrum* have a lower thermal tolerance than shallow colonies, as they suffered partial mortality (i.e., tissue loss) at 24 °C. Nevertheless, we found that they can withstand temperatures that rarely (12 °C and 18 °C) or do not occur yet (21 °C) at 60 m depth (Additional file 1: Fig. S1) [31, 57], as no signs of tissue loss were detected after two months of exposure to temperatures ranging from 12 to 21 °C. In addition, visual observations showed that the polyps remained open throughout the experiment and that *Artemia salina* prey were ingested normally, indicating that the feeding ability of *C. rubrum* was also not affected by the changes in temperature. This may explain why the corals’ energy reserves remained stable, particularly because corals were fed with *Artemia salina* nauplii twice a week. The capability of *C. rubrum* to continue feeding despite large temperature changes may have contributed to its heat tolerance, as has previously been shown for tropical scleractinian corals [81].

To our knowledge, only one study has exposed *C. rubrum* colonies from deep populations (100 m depth) to 25 °C, rapidly rising the temperature from 15 to 25 °C in 48 h, and observed tissue loss after a few days [35]. In our study where temperatures were gradually increased over 36 days, all corals showed tissue loss after two weeks at 24 °C, along with a significant increase in *TNFR1* gene expression. TNF receptors are known regulators of the apoptosis cascade [82–84]. They initiate signal transduction pathways leading to caspase activation and apoptosis in metazoans [83, 84], and play a critical role in regulating immune and stress responses [85]. Besides, corals can rapidly upregulate *TNFR* genes following heat stress [83, 86, 87]. Heat shock protein 70 (HSP70) is also rapidly induced during heat stress to maintain protein structure and function, and to promote cellular repair processes and tolerance in corals, including *C. rubrum* [34, 83, 88]. Yet, the expression of *HSP70* at 24 °C remained at a control level (similar to 15 °C) in our study. This may be due to sampling after two weeks of exposure, thereby failing to capture the expressional changes that often occur at the initial phase of a stress response [89, 90]. Tissue loss was also not related to a decrease in the protein, lipid and carbohydrate reserves, suggesting that biweekly feeding may have helped corals maintain energy reserves under these conditions.

Finally, our results showed an increase of the sclerite content in the coral tissues at 21 °C and 24 °C compared to 15 °C control conditions, which may be due to temperature-dependent enhanced activities of enzymes implicated in coral calcification [32, 91]. Although the lethal temperature threshold between 21 and 24 °C cannot be determined, our results suggest that populations of *C. rubrum* at 60 m depth are likely to survive temperature anomalies between 21 and 24 °C. Although this thermal threshold can be reached in shallow waters, the likelihood of it occurring below 60 m depth remains low, at least within the next few decades [30, 31].

### The microbiota of *C. rubrum* remains preserved over a wide range of temperatures

Bacteria from the Spirochaetaceae family dominated the bacterial communities associated with mesophotic *C. rubrum* (relative abundance of 65% on average in the control 15 °C conditions), which thus share a common feature with its shallow conspecifics, in which Spirochaetaceae account for 42–72% of the bacterial communities depending on location and season [50, 51]. While Endozoicomonadaceae dominate the bacterial communities of many hexa- and octocorals [52, 92, 93], the dominance of Spirochaetaceae is characteristic of *C. rubrum* [52]. To our knowledge, only one study investigated the bacterial community composition of mesophotic red coral colonies, and found a low proportion (< 10%) of Spirochaetaceae [25]. Although this contrasts with our findings, these colonies were collected during the 2011 MHW and the thermal anomalies may thus have induced alterations in the red coral microbiome.

We did observe shifts in the bacterial community composition of red corals from 12 to 21 °C. This could mainly be explained by changes in the relative abundance of bacterial phylotypes already present in the microbiome of the control colonies that were kept at 15 °C. Our results suggest that these compositional changes did not compromise the integrity of the red coral holobiont and may be related to a direct effect of temperature on certain bacterial symbionts rather than the host response to temperature change. Such variations are similar to the seasonal patterns observed in shallow colonies [50, 51]. Moreover, minor changes in the microbiota are commonly observed when corals are exposed to environmental changes that do not pose significant stress to the host [43, 48, 94]. It would have been interesting to return corals to the control temperature of 15 °C and investigate if there is a dynamic microbiome restructuring, indicating that *C. rubrum* exhibits microbiome acclimation and is a ‘microbiome conformer’ [95]. Besides, microbes whose relative abundance did not change under the different thermal conditions may have also contributed to the tolerance of the coral holobiont to the different thermal anomalies, although it was not possible to determine this in our study. To evaluate if the symbionts contribute to the host thermal tolerance, further investigations identifying the functional changes occurring within the microbiota under the different temperature regimes through a combination of metagenomics and metatranscriptomics approaches are needed.

At 18 °C and 21 °C, the relative abundance of Vibrionaceae significantly increased in the microbiomes. Some *Vibrio sp*. have been shown to be pathogenic for corals [96, 97]. The fact that these *Vibrio* sp. did not induce tissue necrosis might be explained by the absence of expression of the virulence factors at temperatures below 23 °C. Indeed, a number of Mediterranean pathogenic Vibrionaceae induce tissue necrosis when temperatures reach 23 °C [97–99] and/or when they reach a certain cell concentration (i.e., pathogenicity activated through quorum sensing) [96].

At 24 °C, dysbiosis was observed in the bacterial community of *C. rubrum* while the host was in poor state of health and suffering tissue loss, consistent with previous observations made on other coral species [48, 100–102]. The relative abundance of the dominant Spirochaetaceae symbionts was reduced from 66% at 15 °C to 8% at 24 °C. A similar relative abundance was observed in the microbiome of mesophotic red coral colonies during the 2011 heat wave [25], supporting the hypothesis that the Spirochaetaceae abundance is an indicator of the health of *C. rubrum*. Concomitantly to the reduced relative abundance of Spirochaetacae, we observed a sharp increase (3000-fold increase from 15 to 24 °C) in the abundance of a *Vibrio sp.,* in colonies with tissue loss. This *Vibrio sp.* might likely be a pathogen because of its high sequence similarity to *V. tubiashii* which is known to degrade the extracellular matrix of bivalves [103]. Other ASVs putatively pathogens such as *Tenacibaculum* sp. (ASV56) [104–106] also increased in *C. rubrum* colonies between 15 and 24 °C. Previous studies have linked changes in the microbiome and physiological stress to coral mortality [25, 102, 107–109], and the sharp increase in potential pathogens observed here and the suitable temperature for the activation of the virulence factors, may have been responsible for the tissue loss on all colonies. The upregulation of the *TNFR1* gene, implicated in the regulation of the immune system [85, 87], is also consistent with an immune or stress response of the host to a putative pathogen infection at 24 °C.

## Conclusion

MHWs in the Mediterranean Sea have increased in frequency, intensity and length in recent decades, with seasonality in their occurrence between the photic and mesophotic zone [9, 31, 57]. While MHWs are observed at the surface between July and September, they occur from mid-October to mid-December at depths of 55–60 m [57]. The projected temperature change by the end of the century ranges from 0.81 to 3.6 °C in the upper layer (0–150 m), depending on the emission scenario and the model used [30, 57, 110]. The temperature at 60 m depth where *C. rubrum* was collected was around 14–15 °C, except in fall where warmer temperatures, which can reach transient (less than a week) peaks of 19 °C, were observed over the last two years. We thus can expect in the worst-case scenario (+ 3.6 °C) annual temperatures of 18.6 °C with transient peaks at 22.6 °C by the end of the century. In this study, we found that mesophotic populations of *C. rubrum* could withstand temperatures as high as 21 °C for two months without signs of physiological impairment or dysbiosis. While tissue loss was observed within two weeks of exposure at 24 °C in the present study, the mesophotic zone is unlikely to reach such a temperature in the near future. Although more research is needed, the mesophotic zone may be thermal refugia for *C. rubrum* and possibly other coral species with large depth distributions, a concept (deep reef refugia hypothesis, DRRH) introduced by Bongaerts et al. [111] for mesophotic tropical reefs, that has since been debated [112]. Further studies exposing corals to acute short-term stress (one or a few days, before reaching mortality) are however needed to investigate both the damage caused by transient exposure to damaging temperatures (> 21 °C) and the ability of the host to recover after the stress has subsided. Finally, our results shed new light on the holobiont response of *C. rubrum* to thermal stress, and suggest that Spirochaetaceae may play an important role in the coral’s overall health. To fully understand the function and stress resistance of the red coral holobiont, future investigations should focus on elucidating the precise role of Spirochaetaceae symbionts.

### Supplementary Information

**Additional file 1:** Supplementary figures and tables.

**Additional file 2:** Metadata and statistical tests.

**Additional file 3:** ASV table.

**Additional file 4:** ASV taxonomy.

**Additional file 5:** ASV sequences.

**Additional file 6:** ASV raw abundances per sample.

## Data Availability

The raw amplicon sequences and accompanying metadata have been deposited in the National Center for Biotechnology Information Sequence Read Archive under the project number PRJNA967137.
