# Supplementary material for: Holobiont responses of mesophotic precious red coral Corallium rubrum to thermal anomalies
Source: Environ Microbiome. 2023 Aug 14;18:70. doi: 10.1186/s40793-023-00525-6 (PMC10424431; doi:10.1186/s40793-023-00525-6)
Supplement: Supplementary file 1 — Additional file 1: Supplementary figures and tables. [file 40793_2023_525_MOESM1_ESM.docx]

Holobiont responses of mesophotic precious red coral *Corallium rubrum* to thermal anomalies

Romie Tignat-Perrier^1,2^, Jeroen A.J.M. van de Water^1,2,3^, Denis Allemand^4^, Christine Ferrier-Pagès^2^

**Affiliations:**

^1^Unité de Recherche sur la Biologie des Coraux Précieux CSM - CHANEL, Centre Scientifique de Monaco, 8 Quai Antoine 1^er^, MC 98000, Monaco, Principality of Monaco

^2^Coral Ecophysiology Laboratory, Centre Scientifique de Monaco, 8 Quai Antoine 1^er^, MC 98000, Monaco, Principality of Monaco

^3^Department of Estuarine & Delta Systems, Royal Netherlands Institute for Sea Research, Korringaweg 7, 4401 NT, Yerseke, The Netherlands

^4^Centre Scientifique de Monaco, 8 Quai Antoine 1^er^, MC 98000, Monaco, Principality of Monaco

**Running title:** Response of deep-dwelling colonies of *Corallium rubrum* to thermal anomalies

**Keywords:** Red coral, *Corallium rubrum*, Octocoral, Gorgonian, Thermal stress, Bacterial communities, Physiology, Holobiont, Gene expression, *16S rRNA* gene sequencing

**Supplementary Figures**


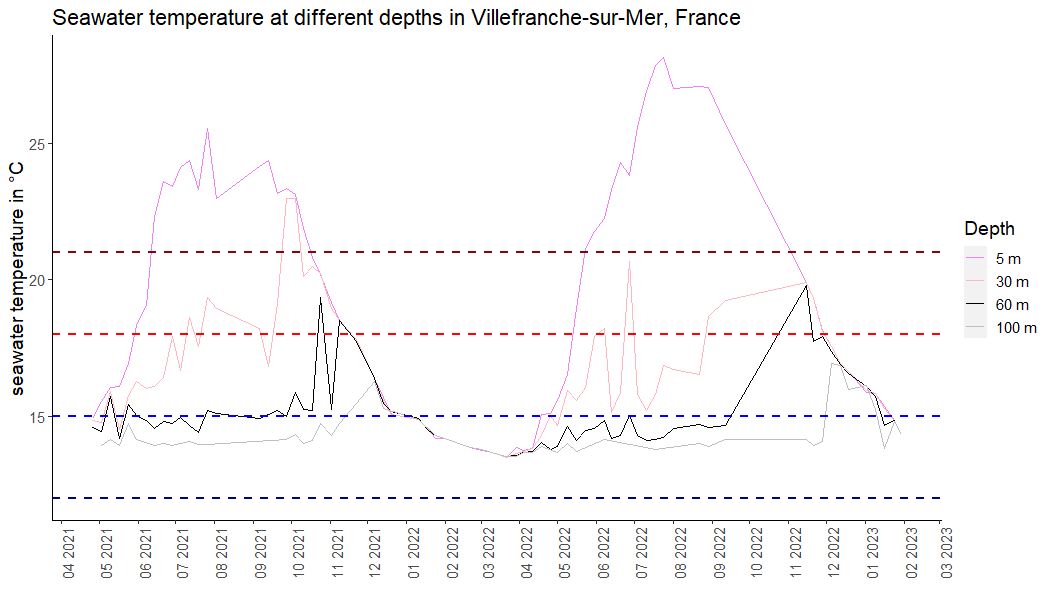


18 °C

21 °C

12 °C

**Figure S1.** Seawater temperature at 5 m (pink), 30 m (orange), 60 m (black) and 100 m (grey) depth from April 2021 to February 2023 in Villefranche-sur-Mer, France. Weekly data obtained from <https://www.somlit.fr/>. A data gap is present between mid-September and November 2022.


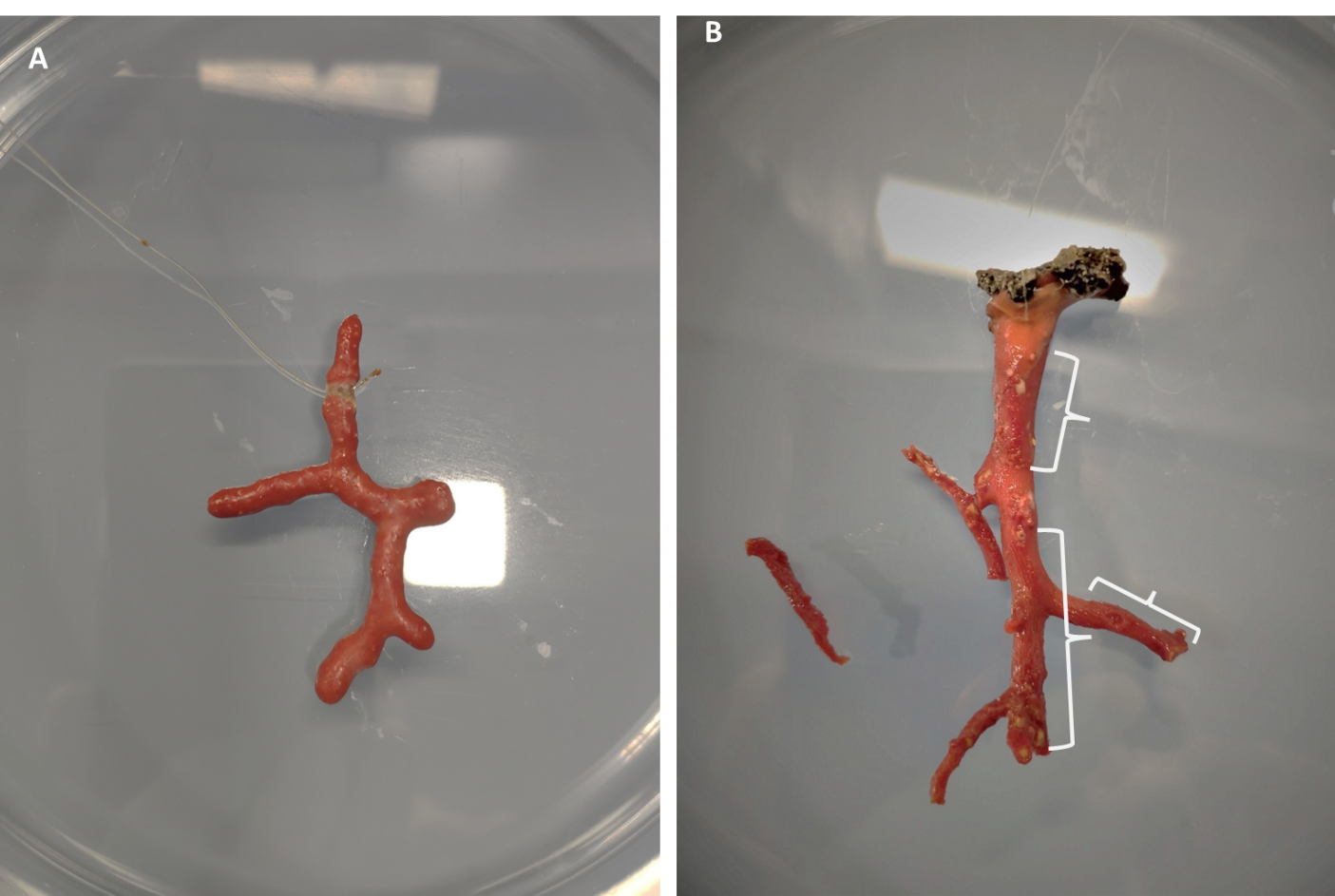


**Figure S2.** Morphological aspect of red coral colonies under the 15 °C (**A**) and 24 °C (**B**) thermal conditions after two weeks of exposure. The white brackets on (**B**) indicate skeleton without tissue.


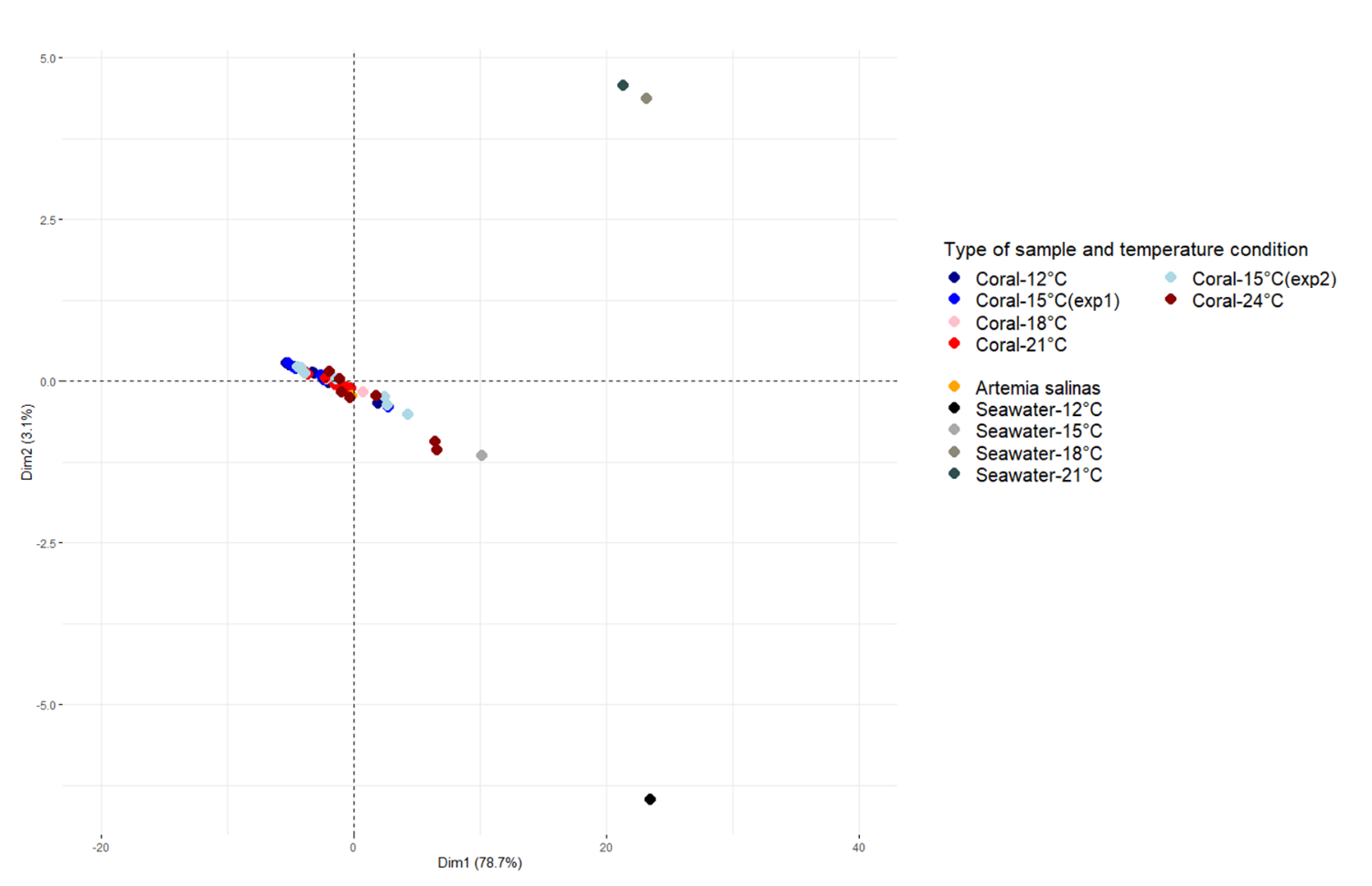


**A**


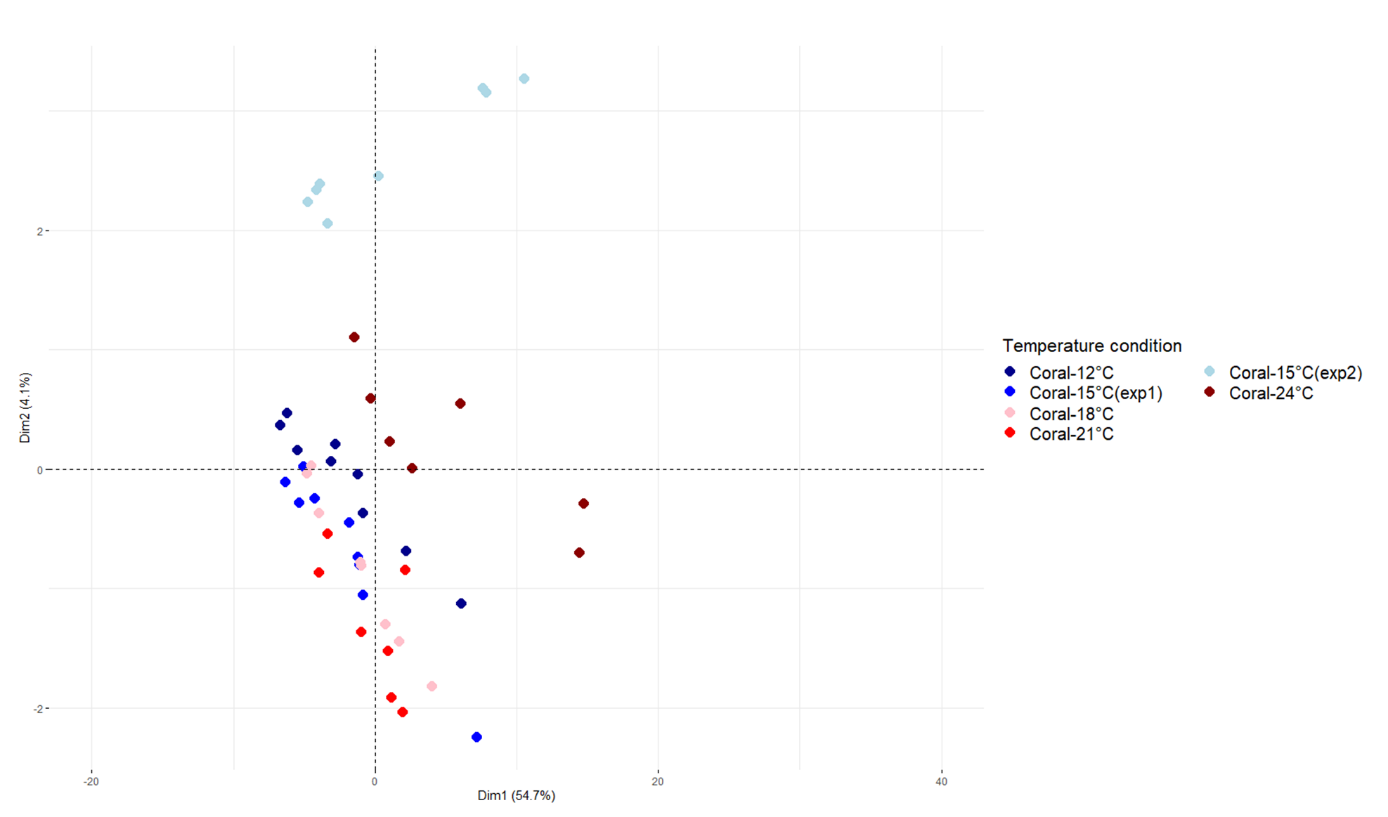


**B**

**Figure S3.** Distribution of the samples based on the bacterial community structure. Principal Component analysis (PCA) on the Aitchison distance matrix based on the structure of the bacterial community (ASV level) associated with the red coral exposed to the different thermal conditions, as well as *Artemia salina* and in seawater. (**A**) PCA showing the distribution of the *Artemia salina*, seawater and coral samples (Experiment 1: 12 °C, 15 °C(exp1), 18 °C and 21 °C and Experiment 2: 15 °C(exp2) and 24 °C); (**B**) PCA showing the distribution of the coral samples (Experiment 1 and 2).


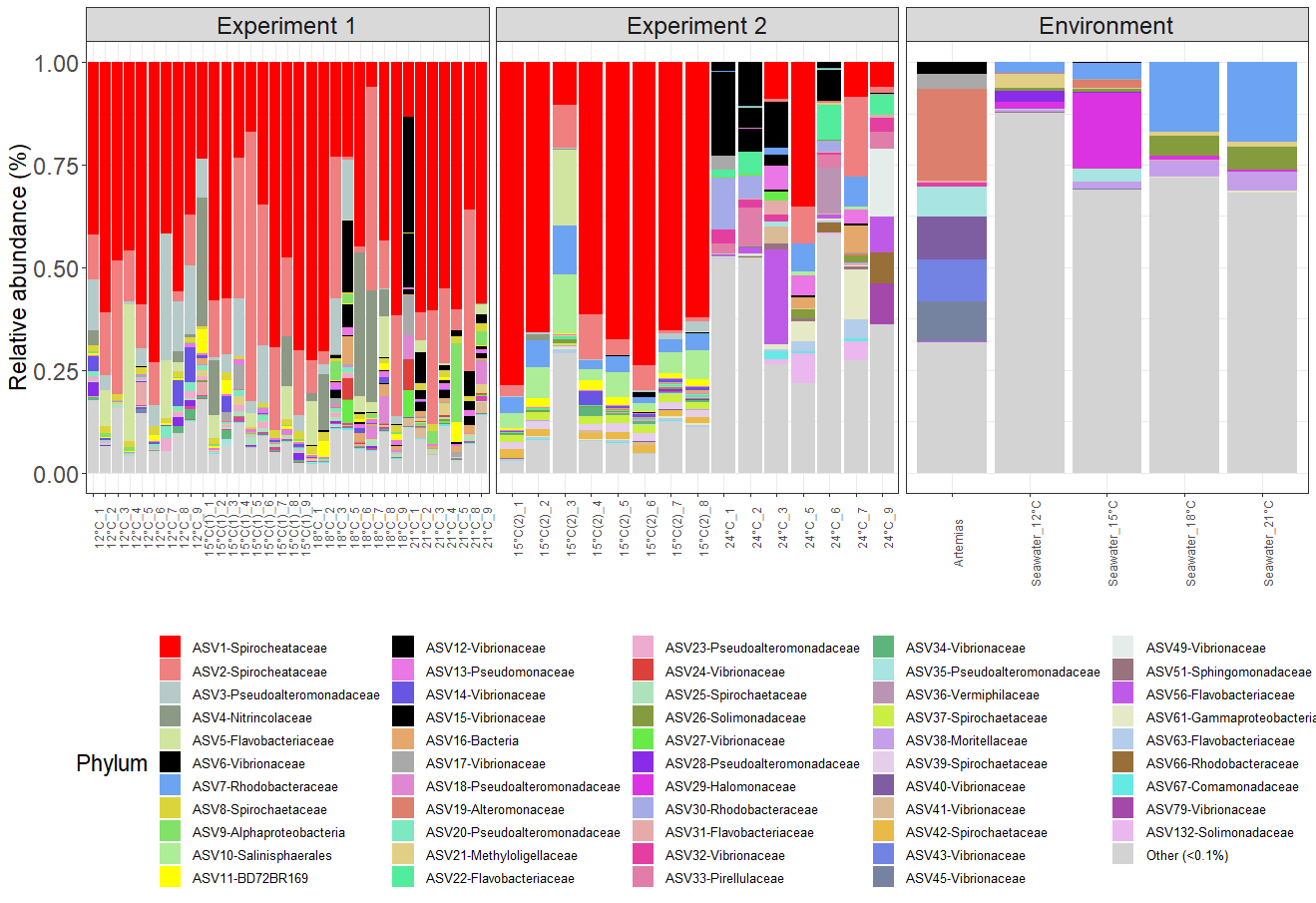


Other (<0.1)

**Figure S4.** Relative abundance of the most abundant bacterial ASVs in the different samples. Relative composition of the most abundant ASVs composing the bacterial community associated with the red coral colonies exposed to the different thermal conditions (**A**- Experiment 1 and **B**- Experiment 2), and the seawater and *Artemia salina* bacterial community (**C**).


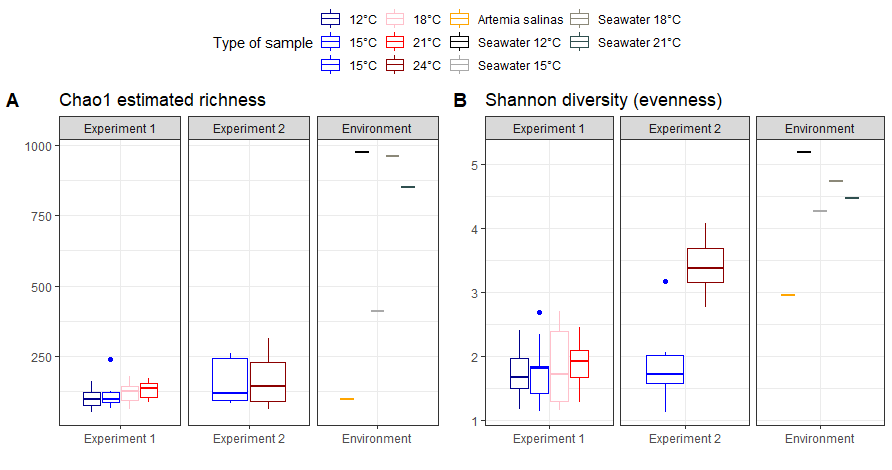


**Figure S5.** Chao1 estimated richness (**A**) and Shannon diversity (**B**) of the bacterial community associated with the red coral colonies exposed to the different thermal conditions, as well as the *Artemia salina* and seawater samples.


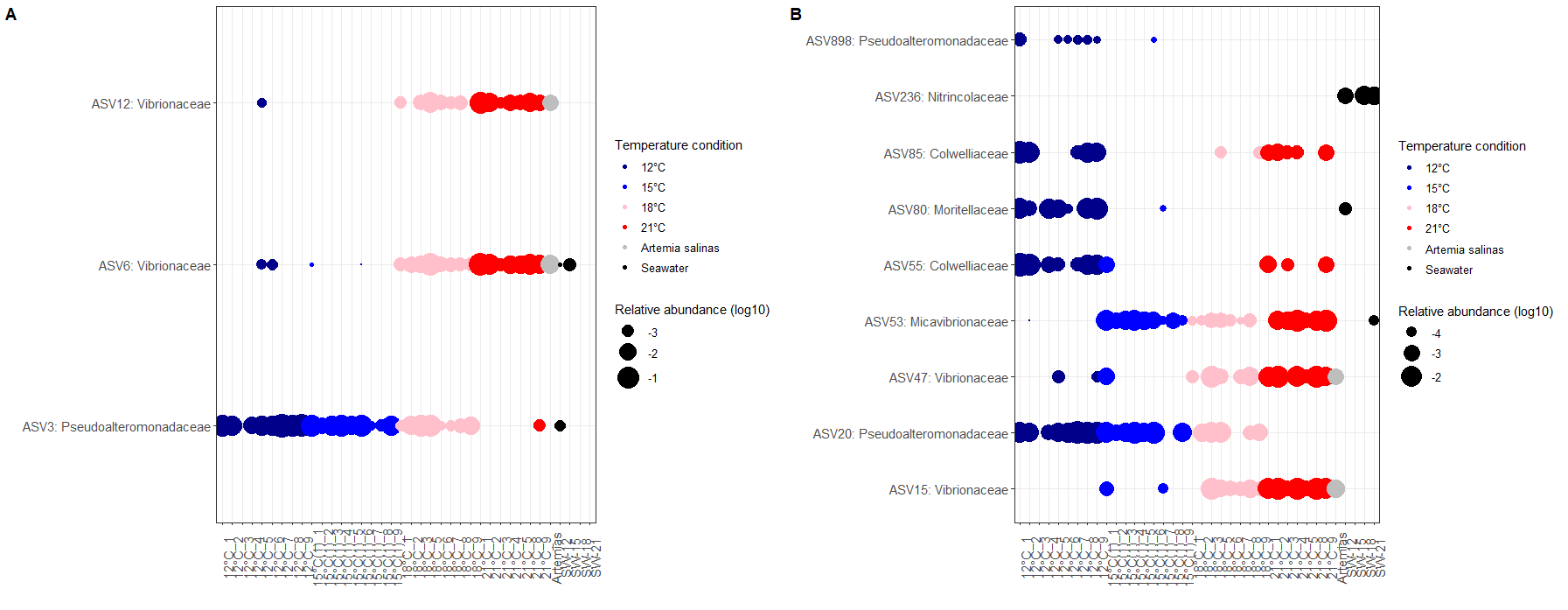


**Figure S6.** Bubble plots of the ASVs whose relative abundance significantly changed within the microbiota of the red coral colonies between the thermal conditions (**Experiment 1**). (**A**) Log10-
transformed relative abundance of the ASVs that represent < 1 % of the community; (**B**) Log10-
transformed relative abundance of the ASVs that represent ≥ 1 % of the community at least in one of the thermal condition.

**
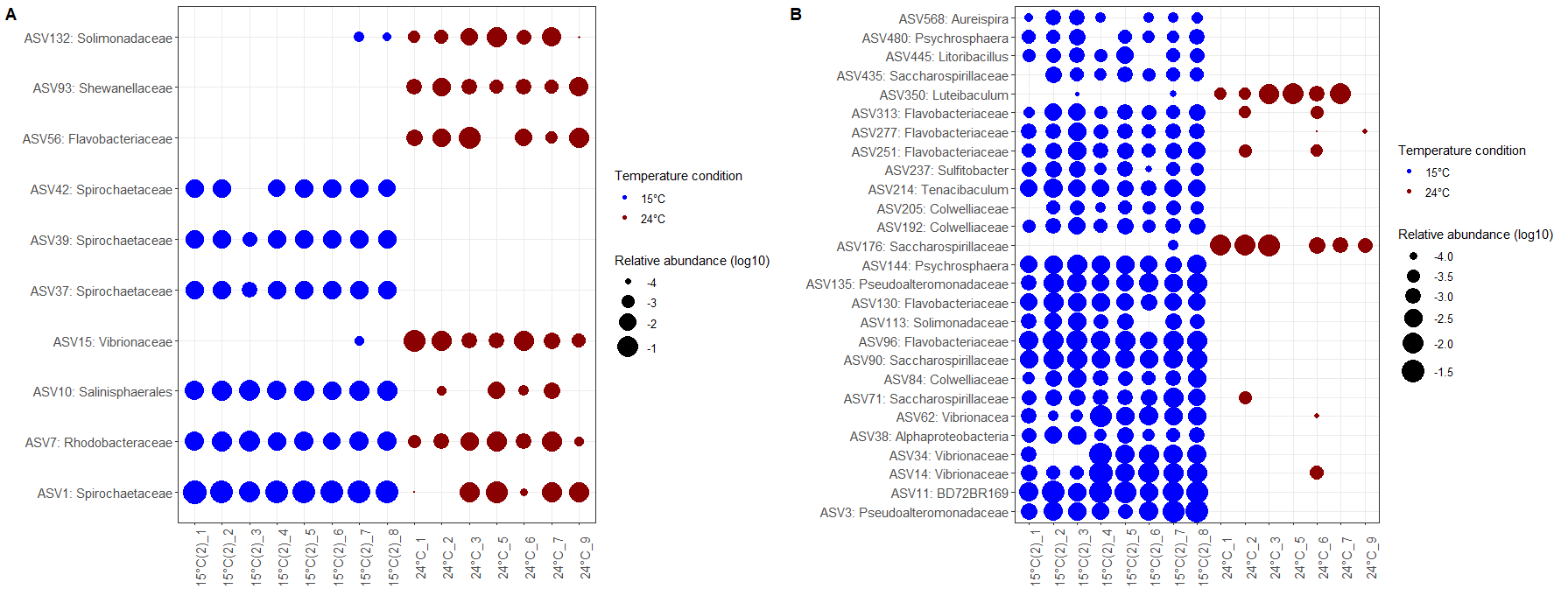
**

**Figure S7.** Bubble plots of the ASVs whose relative abundance significantly changed within the microbiota of the red coral colonies between the thermal conditions (**Experiment 2**). (**A**) Log10-
transformed relative abundance of the ASVs that represent < 1 % of the community; (**B**) Log10-
transformed relative abundance of the ASVs that represent ≥ 1 % of the community at least in one of the thermal condition.

**Supplementary Tables**

**Table S1.** Primer sequences and annealing temperatures for the qPCR analyses.


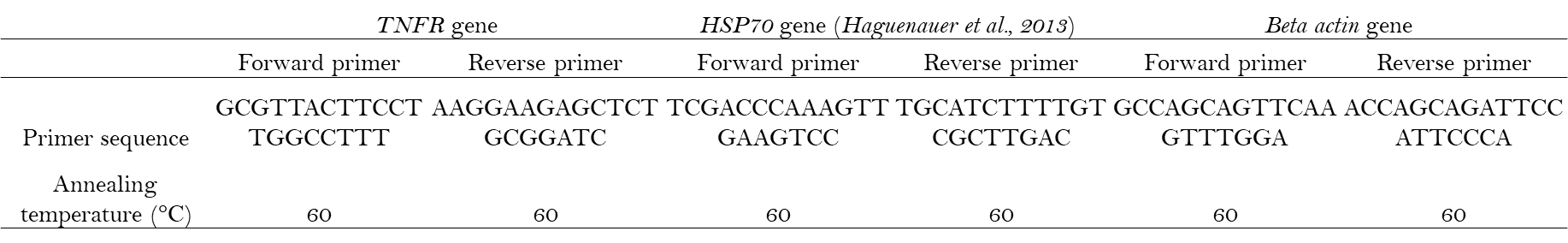


**Table S2.** Number of reads and sequences throughout the DADA2 pipeline for each sample.

| Sample | Paired input reads | Paired filtered reads | Merged sequences | Non-chimeric sequences |
| --- | --- | --- | --- | --- |
| CR-12-1 | 69080 | 55042 | 54403 | 50562 |
| CR-12-2 | 91130 | 73789 | 73437 | 72615 |
| CR-12-3 | 114188 | 90226 | 89731 | 88127 |
| CR-12-4 | 80179 | 57142 | 56902 | 56400 |
| CR-12-5 | 108240 | 85804 | 85125 | 84002 |
| CR-12-6 | 101139 | 74451 | 74192 | 73181 |
| CR-12-7 | 110364 | 90881 | 90494 | 89615 |
| CR-12-8 | 104948 | 84924 | 84498 | 83209 |
| CR-12-9 | 72014 | 57172 | 56685 | 53757 |
| CR-15-1 | 92375 | 71807 | 70521 | 69320 |
| CR-15-2 | 90411 | 72029 | 71760 | 70455 |
| CR-15-3 | 69133 | 46679 | 46384 | 45660 |
| CR-15-4 | 30077 | 22641 | 22159 | 19177 |
| CR-15-5 | 104277 | 71901 | 71253 | 69471 |
| CR-15-6 | 95588 | 73615 | 72995 | 70825 |
| CR-15-7 | 113930 | 88941 | 88657 | 87019 |
| CR-15-8 | 61918 | 48330 | 47604 | 46076 |
| CR-15-9 | 98419 | 79541 | 79154 | 77261 |
| CR-18-1 | 108891 | 89113 | 88722 | 86793 |
| CR-18-2 | 76090 | 53858 | 53426 | 52771 |
| CR-18-3 | 94012 | 71327 | 70379 | 65672 |
| CR-18-5 | 90631 | 66327 | 65382 | 60751 |
| CR-18-6 | 100500 | 79992 | 78415 | 75273 |
| CR-18-7 | 103210 | 76482 | 74583 | 69383 |
| CR-18-8 | 101257 | 76629 | 74881 | 65363 |
| CR-18-9 | 92487 | 72132 | 71282 | 68168 |
| CR-21-1 | 111004 | 89092 | 88556 | 85430 |
| CR-21-2 | 65704 | 45053 | 44389 | 43222 |
| CR-21-3 | 103619 | 80706 | 80205 | 78517 |
| CR-21-4 | 97808 | 71797 | 71152 | 67924 |
| CR-21-5 | 122538 | 97774 | 96781 | 94888 |
| CR-21-8 | 109858 | 83498 | 83055 | 80033 |
| CR-21-9 | 76269 | 50165 | 49262 | 42273 |
| Artemia salina | 72585 | 54861 | 53848 | 50787 |
| CR-2-15-1 | 70361 | 53075 | 52858 | 52815 |
| CR-2-15-2 | 37799 | 28751 | 28476 | 28331 |
| CR-2-15-3 | 69134 | 51736 | 51094 | 50906 |
| CR-2-15-4 | 59621 | 40072 | 39673 | 38966 |
| CR-2-15-5 | 47665 | 35988 | 35778 | 35675 |
| CR-2-15-6 | 79525 | 59430 | 59107 | 58832 |
| CR-2-15-7 | 76795 | 58846 | 58350 | 58061 |
| CR-2-15-8 | 95564 | 72517 | 71827 | 70993 |
| CR-2-24-1 | 58946 | 43366 | 40661 | 33965 |
| CR-2-24-2 | 63312 | 47018 | 45828 | 45171 |
| CR-2-24-3 | 2842 | 1991 | 1838 | 1738 |
| CR-2-24-5 | 3620 | 2579 | 2420 | 2353 |
| CR-2-24-6 | 86746 | 61854 | 59719 | 58968 |
| CR-2-24-7 | 28478 | 21076 | 20740 | 20437 |
| CR-2-24-9 | 49858 | 37020 | 35971 | 31301 |
| Negative control | 9754 | 7400 | 7392 | 7392 |
| SW-12 | 127859 | 94957 | 76051 | 69132 |
| SW-15 | 79546 | 49172 | 39872 | 32831 |
| SW-18 | 113639 | 83158 | 69548 | 63446 |
| SW-21 | 124474 | 87282 | 69562 | 58272 |
